# Alpha-enolase (ENO1) controls alpha v/beta 3 integrin expression and regulates pancreatic cancer adhesion, invasion, and metastasis

**DOI:** 10.1186/s13045-016-0385-8

**Published:** 2017-01-13

**Authors:** Moitza Principe, Simone Borgoni, Mariafrancesca Cascione, Michelle Samuel Chattaragada, Sammy Ferri-Borgogno, Michela Capello, Sara Bulfamante, Jennifer Chapelle, Francesca Di Modugno, Paola Defilippi, Paola Nisticò, Paola Cappello, Chiara Riganti, Stefano Leporatti, Francesco Novelli

**Affiliations:** 1 Department of Molecular Biotechnology and Health Sciences, University of Turin, Turin, Italy; 2 Center for Experimental Research and Medical Studies (CeRMS), Azienda Universitaria Ospedaliera Città della Salute e della Scienza di Torino, Via Santena 5, 10126 Turin, Italy; 3 Dipartimento di Matematica e Fisica “Ennio De Giorgi”, Università del Salento, Lecce, Italy; 4 Euromediterranean Center for Nanomaterial Modelling and Technology (ECMT) of the Consiglio Nazionale delle Ricerche, Istituto Nanoscienze, Lecce, Italy; 5 Molecular Biotechnology Center, University of Turin, Turin, Italy; 6 Regina Elena National Cancer Institute, Rome, Italy; 7 Department of Oncology, University of Turin, Turin, Italy; 8 CNR Nantotec-Istituto di Nanotecnologia, Polo di Nanotecnologia c/o Campus Ecoteckne, Lecce, Italy

**Keywords:** Pancreatic cancer, ENO1, Integrin, Atomic force microscopy, Invasion

## Abstract

**Background:**

We have previously shown that in pancreatic ductal adenocarcinoma (PDA) cells, the glycolytic enzyme alpha-enolase (ENO1) also acts as a plasminogen receptor and promotes invasion and metastasis formation. Moreover, ENO1 silencing in PDA cells induces oxidative stress, senescence and profoundly modifies PDA cell metabolism. Although anti-ENO1 antibody inhibits PDA cell migration and invasion, little is known about the role of ENO1 in regulating cell-cell and cell-matrix contacts. We therefore investigated the effect of ENO1 silencing on the modulation of cell morphology, adhesion to matrix substrates, cell invasiveness, and metastatic ability.

**Methods:**

The membrane and cytoskeleton modifications that occurred in ENO1-silenced (shENO1) PDA cells were investigated by a combination of confocal microscopy and atomic force microscopy (AFM). The effect of ENO1 silencing was then evaluated by phenotypic and functional experiments to identify the role of ENO1 in adhesion, migration, and invasion, as well as in senescence and apoptosis. The experimental results were then validated in a mouse model.

**Results:**

We observed a significant increase in the roughness of the cell membrane due to ENO1 silencing, a feature associated with an impaired ability to migrate and invade, along with a significant downregulation of proteins involved in cell-cell and cell-matrix adhesion, including alpha v/beta 3 integrin in shENO1 PDA cells. These changes impaired the ability of shENO1 cells to adhere to Collagen I and IV and Fibronectin and caused an increase in RGD-independent adhesion to vitronectin (VN) via urokinase plasminogen activator receptor (uPAR). Binding of uPAR to VN triggers integrin-mediated signals, which result in ERK1-2 and RAC activation, accumulation of ROS, and senescence. In shENO1 cancer cells, the use of an anti-uPAR antibody caused significant reduction of ROS production and senescence. Overall, a decrease of in vitro and in vivo cell migration and invasion of shENO1 PDA cells was observed.

**Conclusion:**

These data demonstrate that ENO1 promotes PDA survival, migration, and metastasis through cooperation with integrins and uPAR.

**Electronic supplementary material:**

The online version of this article (doi:10.1186/s13045-016-0385-8) contains supplementary material, which is available to authorized users.

## Background

Pancreatic ductal adenocarcinoma (PDA) is one of the most lethal forms of cancer with a 5-year survival rate of less than 8% [1]. No early detection tests are currently available and, as a result, most patients (80–85%) are not diagnosed until late-stages of the disease, when the cancer has metastasized to other organs [2–4]. In recent years, many proteins have been proposed as new immunological and molecular targets for PDA [5–7] and among these, one of the most promising is alpha-enolase (ENO1) [8].

In PDA and other tumors, ENO1 has a multifunctional role depending on its localization [8, 9]. In addition to its well-known enzymatic function during glycolysis, ENO1 acts as a plasminogen receptor on the cell surface [8, 10] promoting metastatic cancer invasion [11–15]. We have previously demonstrated that the injection of adenovirus expressing cDNA coding for monoclonal antibody that block the binding of ENO1 with plasminogen-inhibited metastases formation of PDA cells in vivo [12]. Many metabolic enzymes, including ENO1, are known to interact with cytoskeletal proteins (e.g., F-actin, tubulin), and these associations provide ATP to promote the migration of tumor cells [16, 17]. Although the roles of ENO1 as a glycolytic enzyme [18, 19] and as a plasminogen receptor [12, 20] have been well characterized, its role in the regulation of cytoskeleton reorganization, particularly in PDA cells, has not been fully clarified. Integrins regulate adhesion-dependent growth and invasion of tumor cells and the integrin alpha v/beta 3 has been reported as a mediator of anchorage independence [21], and its expression has been associated with an aggressive form of disease in different human tumors including PDA [22–27]. In this study, we employed biochemical and functional approaches to investigate molecules involved in cell adhesion and migration of ENO1-silenced (shENO1) PDA cells. AFM (atomic force microscopy) was employed to investigate the nanostructural properties of shENO1 PDA cells. In addition, as cell adhesion, survival and migration are dependent on integrin binding to the extracellular matrix (ECM), and subsequent signals, the roles of alpha V/beta 3 and alpha 5/beta 1 integrins, as well as uPAR (an ECM receptor) were evaluated in shENO1 PDA cells. Moreover, the impact of ENO1 silencing in the in vitro and in vivo invasion and spreading of PDA cells was evaluated. Results from this study indicated that ENO1, by cooperating with integrins and uPAR, is a key regulator of cell survival, adhesion, and motility in PDA.

## Methods

### Cell culture

Human pancreatic cancer cell lines used were: CFPAC-1 (from ECACC), PT45, and T3M4. Cell lines were cultured in DMEM (Lonza, Milan, Italy) supplemented with 10% FBS (Lonza), L-Glutamine (GE Healthcare, Milan, Italy) and 50 μg/ml of gentamicin (Gentalyn 40 mg/ml, Essex Italia, Segrate, MI, Italy) at 37 °C in a 5% CO_2_ atmosphere. Cells were detached using a 1 mM EDTA solution in phosphate-buffered saline (PBS).

### Silencing of ENO1 in PDA cell lines

ENO1 was silenced in PDA cell lines using a short hairpin RNA (shRNA), as previously described [12].

### Quantitative PCR

Total RNA was extracted using the RNeasy Mini kit (Qiagen, Milan, Italy) and reverse transcription was performed from 1 μg of total RNA using the iScript cDNA synthesis kit (BioRad, Segrate, MI, Italy), according to the manufacturer’s instructions. Quantitative RT-PCR was performed using SYBR Green dye (Life Technologies, Monza, Italy) on a Thermal iCycler (BioRad). PCR reactions were performed in triplicate, and the relative amount of cDNA was calculated by the comparative CT method using β-actin RNA sequences as a control. Data are represented as mean ± SEM (standard error of the mean) of three independent experiments. Oligonucleotide primer sequences for Sybr Green qRT-PCR are in the Supplementary Materials and Methods (Additional file 1: Table S1).

### CAT microscopy

Images of PDA cells were acquired by using CAT (confocal-atomic force-total internal reflection fluorescence) microscopy, which is a combination of an advanced scanning probe microscope (Bioscope Catalyst, Bruker Inc. USA), a confocal microscope (LSM 700, Zeiss Germany), and a total internal reflection fluorescence microscope (Laser TIRF 3, Zeiss). These devices were mounted on an inverted microscope (Zeiss ObserverZ1, Zeiss). In this study, CAT was used to evaluate topography on living cell surfaces through AFM and internal organization of fibers into cytoskeleton by confocal LSM.

### AFM imaging

For topography acquisition, cells were cultured in plastic Petri dishes (Corning by Sigma-Aldrich), as previously described, and were left to grow until 70–80% confluent. Immediately before performing measurements, cells were washed with PBS solution and the medium was replaced with 5 ml of Lebovitz culture medium (L15, Sigma-Aldrich). Topographic and deflection images were acquired on living cells for up to 2 h in contact mode, as previously described [28]. By using V-shaped silicon nitride cantilevers (MSNL-10 VEECO, USA), probes were chosen on the basis of a low range of elastic constant value (nominal constants from 0.01 to 0.03 N*nm^−1^). Topographic images were acquired at high resolution (512*512 points*line) on a 50*50 μm^2^ area, and were used to calculate the roughness values using the Nanoscope Analysis Software. Prior to calculating the roughness, all images were firstly treated with a second order planefit, then with a second order flattening for deleting every bow and minimizing sample three-dimensionality effects. The roughness was evaluated on 25 areas of 1 μm*1 μm, separately acquired on nuclear regions and cytoplasmatic regions. The mean value and its standard deviation were obtained in this way.

### Confocal microscopy

For the confocal microscopy study, shCTRL and shENO1 cells were seeded at 4 × 10^4^ cells/chamber well and were incubated at 37 °C in a humidified 5% CO_2_ atmosphere. After 24 h, the medium was removed, and cells were washed three times with PBS. Finally, cells were fixed with 4% paraformaldehyde in PBS for 20 min at room temperature and permeabilized with 0.1%Triton X-100 in PBS for 5 min at room temperature. Cells were incubated with anti-ENO1 mAb at a dilution of 1:1000, followed by an Alexa Fluor 488-conjugated goat anti-mouse IgG (H + L) (Life Technologies) at a dilution of 1:250, each for 1 h at room temperature. For actin staining, Phalloidin–Tetramethylrhodamine B isothiocyanate (TRITC) (Sigma-Aldrich) was used at a dilution of 1:1000 for 30 min at room temperature. Cells were then washed with PBS, and samples were covered with a glass slide using Fluoroshiel with DAPI (Sigma-Aldrich) as a mounting medium. DAPI was used for nucleus staining. Samples were kept at 4 °C in the dark until microscopic examination. Confocal acquisitions were performed by using a 100×, 1.46 numerical aperture oil immersion objective. Laser beams with 405, 488, and 555 nm excitation wavelengths were used to detect the blue fluorescence from DAPI, green fluorescence from AlexaFluo 488, and red fluorescence from TRITC, respectively. Finally, confocal data files were processed using ZEN software (Zeiss).

### Adhesion assay

For cell attachment to matrix assays, Vitronectin, Fibronectin, Collagen I, and IV (all from Sigma-Aldrich) were diluted to 10 μg/ml in PBS and adsorbed onto 96-well dishes at 4 °C overnight and then blocked for 2 h at 37 °C with 2% BSA in PBS. Wells were then washed three times with PBS before adding cells. PDA cells, either shCTRL or shENO1, were harvested, centrifuged briefly, then resuspended at a density of 5 × 10^4^ cells/ml in DMEM with 2% FBS. Cells were then seeded onto the coated wells and allowed to adhere for 1 h at 37 °C in the presence or absence of anti-human CD61 (beta3 integrin-Santa Cruz Biotechnology) at a concentration of 10 μg/ml. Non-adherent cells were removed by rinsing with PBS three times. Cells were then fixed with 2% glutaraldehyde (Sigma-Aldrich) in PBS for 20 min and stained with crystal violet (Sigma-Aldrich). Stained cells were washed with PBS, and the dye was solubilized with 10% acetic acid (Sigma-Aldrich) for 5 min on a rocker. Attachment was quantified by measuring the absorbance at 570 nm.

### Western blot analyses

PDA cells were harvested, lysed, resolved, and transferred to nitrocellulose membranes, as previously described [29]. Membranes were incubated overnight at 4 °C with the following antibodies (all diluted in TTBS with 5% BSA): mouse anti-human integrin alpha v (1:500, clone L230 made in-house), mouse anti-human FAK (1:1000, made in-house), mouse anti-human integrin alpha 5 and rabbit anti-human Src (both at 1:1000, Santa Cruz Biotechnology by D.B.A. Italia, Segrate, MI, Italy), mouse anti-human integrin beta 1 (1:500, BD Bioscience, Buccinasco, MI, Italy), mouse anti-human RAC1 (1:1000 Millipore by D.B.A Segrate, MI, Italy), rabbit anti-human ERK1-2 (1:500, GeneTex by Prodotti Gianni, Milan, Italy); rabbit anti-human integrin beta 3, rabbit anti-human uPAR, rabbit anti-human phospho ERK1-2, rabbit anti-human Paxillin, rabbit anti-human phospho Paxillin, rabbit anti-human phospho FAK, rabbit anti-human p38MAPK, rabbit anti-human phospho p38MAPK and rabbit anti-human phospho Src (all 1:1000, Cell Signaling Technology by EuroClone, Pero, MI, Italy).The mouse anti-human ENO1 (clone 72/1 [30]) and rabbit anti-human beta-actin antibody (Sigma Aldrich, Milan, Italy), both at a dilution of 1:2000 in TTBS, were incubated for 1 h at room temperature. Membranes were then washed with TTBS and probed for 1 h at room temperature with an HRP-conjugated anti-mouse IgG (Santa Cruz Biotechnology) or an HRP-conjugated goat anti-rabbit IgG secondary antibody (Sigma Aldrich), accordingly, at a dilution of 1:2000. Immunodetection was carried out by enhanced chemiluminescence using ECL PLUS (GE Healthcare).

### Flow cytometric analysis

A total of 1 × 10^5^ cells were incubated with primary antibody: mouse IgG1 anti-human CD51/61 (alpha v/beta 3 integrin, BD, Milan, Italy), mouse IgG2a anti-human CD41/61 (alpha IIb/beta 3 integrin, BioLegend by Campoverde, Milan, Italy), mouse IgG1 anti-human beta1 integrin (Santa Cruz Biotechnology) or an isotype-matched negative control IgG1 or IgG2a antibody (Ab) accordingly (Dako, Milan, Italy), all at doses of 10 μg/ml for 30 min at 4 °C. After incubation with primary Abs, cells were then incubated with a secondary APC goat anti-mouse IgG Ab (BioLegend) for 20 min at 4 °C. Following this, cells were resuspended in PBS, acquired with a BD Accuri C6 Flow Cytometer (BD) and analyzed using FlowJo 7.5 software.

### RAC GTPase activation assay

To perform the RAC assay, cells were washed twice on ice with PBS and then lysed in 50 mM Tris, 150 mM NaCl, 1% NP40, 10% glycerol, 10 Mm MgCl_2_, and 10 μg/ml each of leupeptin, pepstatin, and aprotinin. Equal amounts of cell extracts were incubated at 4 °C for 1 h with glutathione-coupled Sepharose 4B beads (GE Healthcare) bound to recombinant GST-PAK CRIB domain. Bound proteins were eluted in 2× Laemmli-reducing sample buffer and immunoblotted for RAC1.

### ROS measurement

PDA cells were cultured for 24 h in presence or absence of 10 μg/ml anti-uPAR antibody (clone 3C6, Sigma-Aldrich). ROS measurement was performed as previously described [19].

### Analysis of SA-β-galactosidase activity

For the senescence assay on extracellular matrices, vitronectin, and collagen I (from Sigma-Aldrich) were coated onto 96-well dishes, as described above. 3 × 10^4^ cells/well PDA cells, plus either shCTRL or shENO1, were plated onto coated wells for 72 h in presence or absence of 10 μg/ml anti-uPAR antibody (clone 3C6, Sigma-Aldrich). Images were taken at microscope with ×10 objective. The SA-β-galactosidase activity was analyzed as previously described [19].

### Apoptosis assay

PDA cells were cultured for 48 h in presence or absence of 10 μg/ml anti-uPAR antibody (clone 3C6, Sigma-Aldrich), and apoptotic cells were analyzed using Annexin V Apoptosis Detection Kits (eBioscience by Prodotti Gianni, Milan, Italy).

### Scratch wound healing assay

PDA cells (1×10^5^), plus either shCTRL or shENO1, were seeded into each well of ibidi Culture-Inserts (Ibidi by Giemme, Milan, Italy) and grown to confluence. After 12 h, a cell-free gap of about 500 μm was created after removing the Culture-Insert, and cells were washed twice with serum-free medium to remove any floating cells. Cells which had migrated into the wounded area or protruded from the border of the wound were visualized and photographed under an inverted microscope at each time point over a period of 24 h.

### In vitro chemo-invasion assay

The invasive potential of the PDA cell lines was determined using a modified two-chamber invasion assay, as previously described [12].

### In vivo experiments

ShCTRL or shENO1 CFPAC-1 cell lines were harvested, washed three times, and resuspended in PBS. NOD-SCID IL2Rgamma^null^ (NSG) mice (provided by the animal facility of the Molecular Biotechnology Center, University of Turin, Italy) were injected into the tail vein (i.v.) with 1×10^5^ PDA cells (in 0.1 ml PBS). After 28 days, mice were euthanized, necropsied, and examined for the presence of tumor masses. For the in vivo experiments, five mice were used in each group.

### Tissue samples and histopathology

Tumor masses and main organs of mice were fixed in 4% (v/v) neutral-buffered formalin (Sigma-Aldrich) overnight, transferred to 70% ethanol, followed by paraffin-embedding. For histological analysis, 5-μm formalin-fixed paraffin-embedded tissue sections were cut and stained with hematoxylin-eosin. Tumor/normal tissue ratios were evaluated with ImageJ software.

### Statistical analysis

The Student’s *t* test (GraphPad Prism 5 Software, San Diego, CA) was used to evaluate statistically significant differences in in vitro and in vivo tests. Values were expressed as mean ± SEM.

## Results

### Altered expression of adhesion and cytoskeletal proteins in shENO1 PDA cells

The CFPAC-1 PDA cell line was silenced with a lentivirus that delivered a short hairpin RNA targeting ENO1 3’UTR (shENO1), or a scrambled shRNA (shCTRL) as a control [12]. Previous LC-MS/MS semi-quantitative proteomic analysis using LTQ-Orbitrap on shENO1 CFPAC-1 cells showed significant alterations in the expression of 17 proteins involved in cell adhesion and cytoskeleton organization [19]. Four of these proteins [actin related protein 2/3 complex subunit 4 isoform a (ARPC4), capping protein actin filament muscle Z-line alpha 2 (CAPZA2), secreted phosphoprotein 1 isoform a (SPP1 also named Osteopontin), and breast cancer anti-estrogen resistance 1 (BCAR1 also named p130cas)] were upregulated, and 13 [AHNAK nucleoprotein isoform 1 (AHNAK), anterior gradient protein 2 (AGR2), catenin, delta 1 isoform 1ABC (CTNND1), hypothetical protein LOC64855 isoform 2 (MINERVA), Galectin 3 (LGALS3), catenin alpha 1 (CTNNA1), integrin alpha v isoform 1 precursor (ITGAV), Galectin 4 (LGALS4), Golgi apparatus protein 1 isoform 1 (GLG1), mucin 5AC (MUC5AC), serine or cysteine proteinase inhibitor clade B ovalbumin member 5 (SERPINb5), PDZ and LIM domain 1 (PDLIM1), and cysteine-rich protein 1 intestinal (CRIP1)] were downregulated [19].

**Fig. 1:**
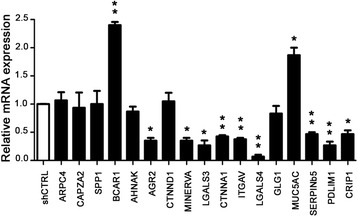
mRNA expression of modulated proteins in CFPAC-1 shENO1 cells. Using real-time PCR, mRNA expression of different proteins was investigated in CFPAC-1 shENO1 cells. Values are expressed as relative expression compared to control cells. A representative of three independent experiments is shown. Data are mean ± SEM. *p < 0.05, **p < 0.01, ***p < 0.001 relative to control cells

Herein, we studied whether the previously observed protein modulation also occurred at the RNA level. Quantitative real-time PCR analysis in shENO1 CFPAC-1 cells indicated that, of the four upregulated proteins, only BCAR1 (p130cas) showed a significant increase in mRNA expression, while the other three proteins had unchanged mRNA expression (Fig. 1). Among the 13 proteins that were downregulated after ENO1 silencing, the expression of mRNA was significantly reduced in nine of them, namely, AGR2, MINERVA, LGALS3, CTNNA1, ITGAV, LGALS4, SERPINSb5, PDLM1, and CRIP1. The mRNA expression was unchanged in three of the remaining four proteins (AHNAH, CTNND1, and GLG1) or was upregulated (MUC5AC) (Fig. 1).

### Morphological and nanostructural modifications in shENO1 PDA cells

**Fig. 2:**
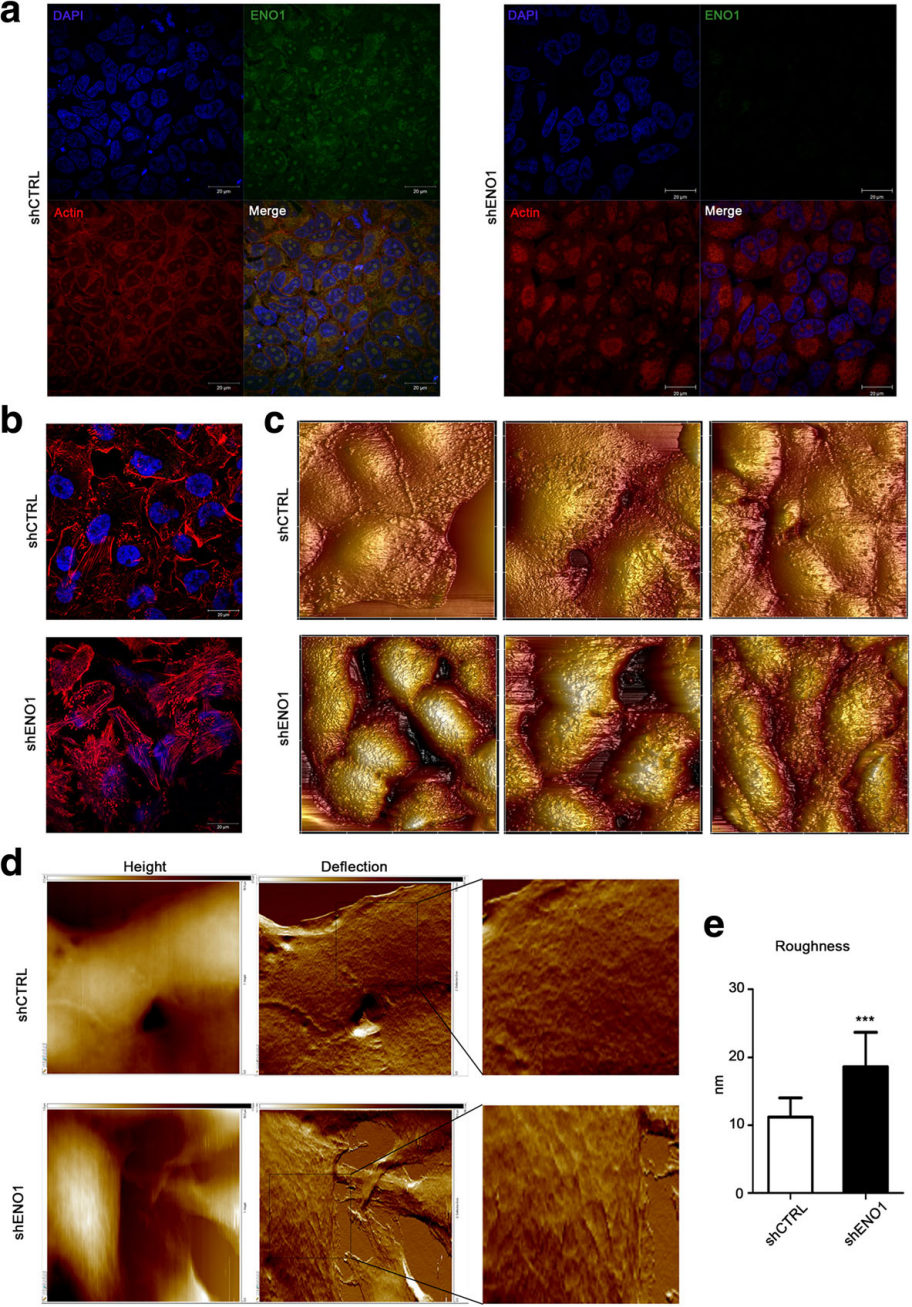
Morphological analysis and roughness measurements of shENO1 cells. **a** shCTRL and shENO1 CFPAC-1 cells stained for nuclei (DAPI, *blue fluorescence*), actin (Phalloidin-TRITC, *red fluorescence*), and ENO1 (anti-ENO1 mAb followed by anti-mouse FITC, *green fluorescence*). Panels represent images taken at the level of nuclei. **b** Images taken at the level of the cell surface. **c** Three-dimensional topographic images of shCTRL and shENO1 CFPAC-1 performed through the AFM technique (three representative images for each group) (scan size: 50 μm; Z scale 4 μm). **d** Topographic images of shCTRL and shENO1 CFPAC-1 performed through the AFM technique. *Left panels*: height parameter; *central panels*: deflection parameter; *right panels*: magnification of deflection panel. **e** Histograms represent roughness analysis. A representative of three independent experiments is shown. Data are mean ± SEM. ***p < 0.001 relative to control cells

Semi-quantitative proteomic and mRNA expression analysis of PDA cells concordantly revealed downregulation of nine cell-ECM adhesion-related proteins after ENO1 silencing (Ref. [19] and Fig. 1), and we therefore investigated the impact of ENO1 silencing on the actin cytoskeleton organization and morphology, by confocal analysis. The majority of ENO1 protein (green fluorescence) in shCTRL cells is localized to the cytoplasm (Fig. 2a left panels), while after ENO1 silencing, green fluorescence was lost, as expected (Fig. 2a right panels). Analyzing actin organization (red fluorescence) showed a loss of fluorescence close to the cell membrane in shENO1 cells, with a concomitant increase of cytoplasmic actin with a perinuclear accumulation. Additional experiments performed with lower cell confluence and with images taken at the level of the apical surface showed that shCTRL cells possess well-defined actin filaments (red fluorescence Fig. 2b upper panel), while shENO1 cells showed a loss of cytoskeleton organization and orientation (red fluorescence Fig. 2b lower panel). These confocal microscopy results were then combined with cell surface analysis in atomic force microscopy (AFM). Three-dimensional AFM highlighted that cell-cell junctions were impaired in shENO1 cells (Fig. 2c lower panels) compared to shCTRL cells (Fig. 2c upper panels). The 2-D AFM analysis of the cell surface showed that shCTRL cells had a smooth and intact cell surface and cell membrane ultrastructural components were uniformly distributed (Fig. 2d upper panels). By contrast, shENO1 cells displayed an altered surface morphology with more evident membrane ultrastructure (Fig. 2d lower panels). Quantification of the nanostructural parameters revealed an increase in the cell surface roughness (Fig. 2e) of shENO1 cells, in line with the modification of the expression of proteins involved in the remodeling of the cytoskeleton and adhesion (Ref. [19] and Fig. 1).

### shENO1 PDA cells display reduced adhesion to fibronectin and collagens and increased adhesion to vitronectin

**Fig. 3:**
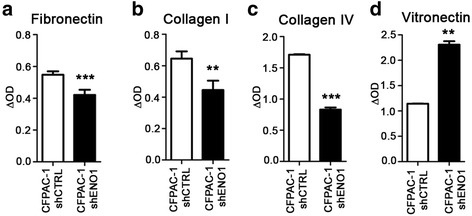
Adhesion ability of ENO1-silenced PDA cells. Adhesive potential of shENO1 and control CFPAC-1 cells was evaluated by culturing cells for 1 h on fibronectin (**a**), collagen I (**b**), collagen IV (**c**)**,** and vitronectin (**d**). Adherent cells were fixed with 2% glutaraldehyde in PBS and visualized by staining with crystal violet. For quantitative analysis, cells were treated with 10% acetic acid and elutes were read with a microplate reader at a wavelength of 570 nm. Results are expressed as ΔOD (optical density) units = (OD substrate adherent cells)–(OD plastic adherent cells). A representative of three independent experiments is shown. Data are mean ± SEM. **p < 0.01, ***p < 0.001 relative to control cells

**Fig. 4:**
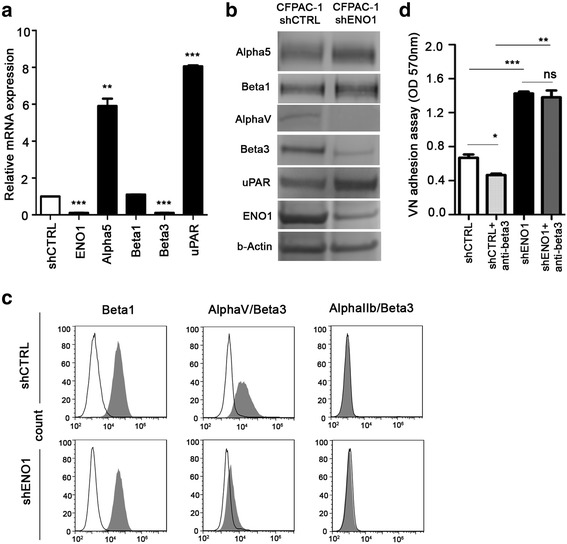
ENO1-silencing modulates ECM-receptor expression. **a** Using quantitative PCR, mRNA coding for different proteins was investigated in shENO1 CFPAC-1 cells. Values are expressed as relative expression compared to control cells. **b** Western blot analysis was carried out to investigate alpha 5, beta 1, alpha v, beta 3, and uPAR expression on total lysates of shCTRL and shENO1 cells. **c** To determine surface expression of integrins, shENO1, or shCTRL cells were incubated with primary antibodies (*gray peak*) against beta 1, alpha v/beta 3, alpha IIb/beta 3, and or isotype-matched control antibody (*empty peak*) and analyzed by flow cytometry. **d** The adhesion ability of shENO1 and shCTRL cells on vitronectin was evaluated in the presence of anti-beta 3 Ab. Adherent cells were fixed with 2% glutaraldehyde in PBS and visualized by staining with crystal violet. For quantitative analysis, cells were treated with 10% acetic acid and elutes were read with a microplate reader at a wavelength of 570 nm. Results are expressed as OD, optical density units. A representative of three independent experiments is shown. Data are mean ± SEM. *p < 0.05, **p < 0.01, ***p < 0.001 relative to control cells

As we showed that the proteins involved in ECM cell adhesion were downregulated in shENO1 cells, we evaluated the adhesive ability of shCTRL or shENO1 PDA cells on fibronectin (FN), collagen I (Col-I), collagen IV (Col-IV), and vitronectin (VN). Compared to shCTRL cells, shENO1 PDA cells (CFPAC-1, PT45 and T3M4) showed a significantly lower adhesion to FN, Col-I, and Col-IV, and a greater adhesion to VN (Fig. 3 and Additional file 1: Figure S1a–b). We then evaluated the expression levels of alpha 5/beta 1 and alpha v/beta 3 complexes, the integrins most involved in binding on these extracellular matrices, in shCTRL and shENO1 cells. Alpha 5 integrin was strongly upregulated by shENO1, both at the mRNA (Fig. 4a) and protein levels (Fig. 4b), while the mRNA, protein, and cell surface levels of beta 1 were unchanged (Fig. 4a–c). mRNA and protein levels of alpha v and beta 3 integrins were decreased by shENO1 (Fig. 1, Fig. 4a and b, respectively). Consistently, the surface expression of the complex alpha v/beta 3, evaluated by a specific Ab that recognized the whole complex, was decreased on shENO1 cells compared to control cells (Fig. 4c). To identify the mechanism involved in the increased VN adhesion mediated by ENO1 silencing, we also analyzed the expression of the other VN receptor alpha IIb/beta 3 integrin. However, no surface expression of this complex was detected in either shENO1 or control cells (Fig. 4c). To understand the involvement of beta 3 integrin in the adhesion to VN, a specific anti-beta 3 Ab was employed. In beta 3-expressing shCTRL cells, the anti-beta 3 Ab significantly decreased the adhesion to VN, while in shENO1 cells that did not express beta 3, it had no effect (Fig. 4d). The increase in adhesion to VN in shENO1 cells with a concomitantly reduced expression of the major integrins usually involved in its binding suggested the involvement of other receptors. As uPAR binds VN through the Somatomedin B (SMB) domain [31, 32], its expression in shENO1 cells was evaluated. Quantitative PCR (Fig. 4a) and western blot (Fig. 4b) analysis showed that uPAR expression was markedly increased in shENO1 cells. These data indicated that the lack of ENO1 caused a decrease in the expression of the main integrins affecting cell adhesion. However, shENO1 cells maintained the ability to bind VN by upregulating uPAR.

### Analysis of signaling pathways in shENO1 PDA cells

**Fig. 5:**
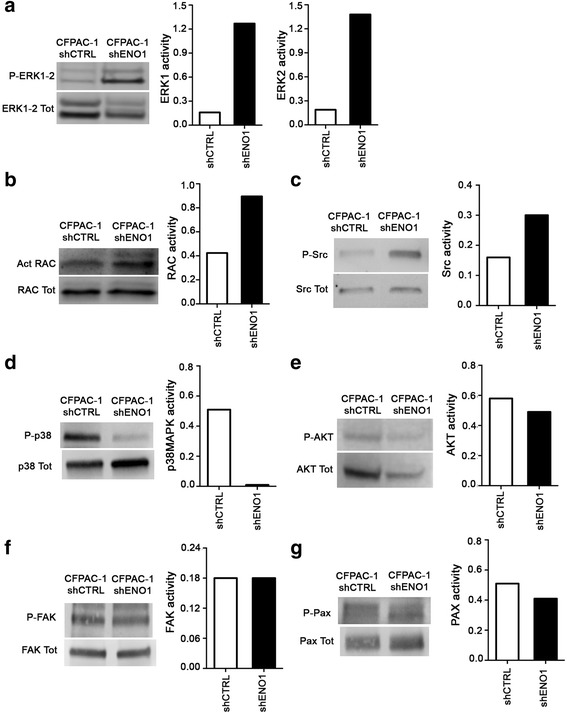
Analysis of uPAR/integrins pathways. Western blot analysis on total lysates of shENO1 and shCTRL CFPAC-1 cells was carried out to investigate levels of (**a**) phospho- and total ERK1-2, (**b**) activated and total RAC, (**c**) phospho- and total Src, (**d**) phospho- and total p38MAPK, (**e**) phospho- and total AKT, (**f**) phospho- and total FAK and (**g**) phospho- and total Paxillin. Histograms represent the ratios between the phosphorylated and total form of each protein. A representative of three independent experiments is shown

It is known that uPAR, despite lacking a cytosolic domain, activates an intracellular signaling pathway through the interaction with beta 1 and beta 3 integrins, independently of their interaction with VN [33–35]. As beta 1 expression was unchanged at the cell surface of shENO1 cells (Fig. 4 b–c), and it can trigger activation of the ERK1-2/RAC pathway [33, 36], the effect of ENO1 silencing on this pathway was analyzed. Western blot analysis revealed a strong increase in activation of both ERK1-2 (Fig. 5a) and RAC (Fig. 5b) in shENO1 cells compared to control cells. To better clarify the effect of ENO1 silencing on integrin-dependent signaling cascades, other key proteins such as the Src, p38MAPK, AKT, FAK, and Paxillin were evaluated. In ENO1-silenced CFPAC-1 cells, the phosphorylation of Src was increased (Fig. 5c) whereas phosphorylation of p38MAPK were downregulated (Fig. 5d). No significant difference was observed in AKT, FAK, and Paxillin (Pax) phosphorylation (Fig. 5e–g). Thus, ENO1 silencing increases Src activation supporting downstream signals via ERK1-2/RAC signaling.

### uPAR blockade in shENO1 cells causes a reduction of ROS and inhibitio﻿n of cell senescence

**Fig. 6:**
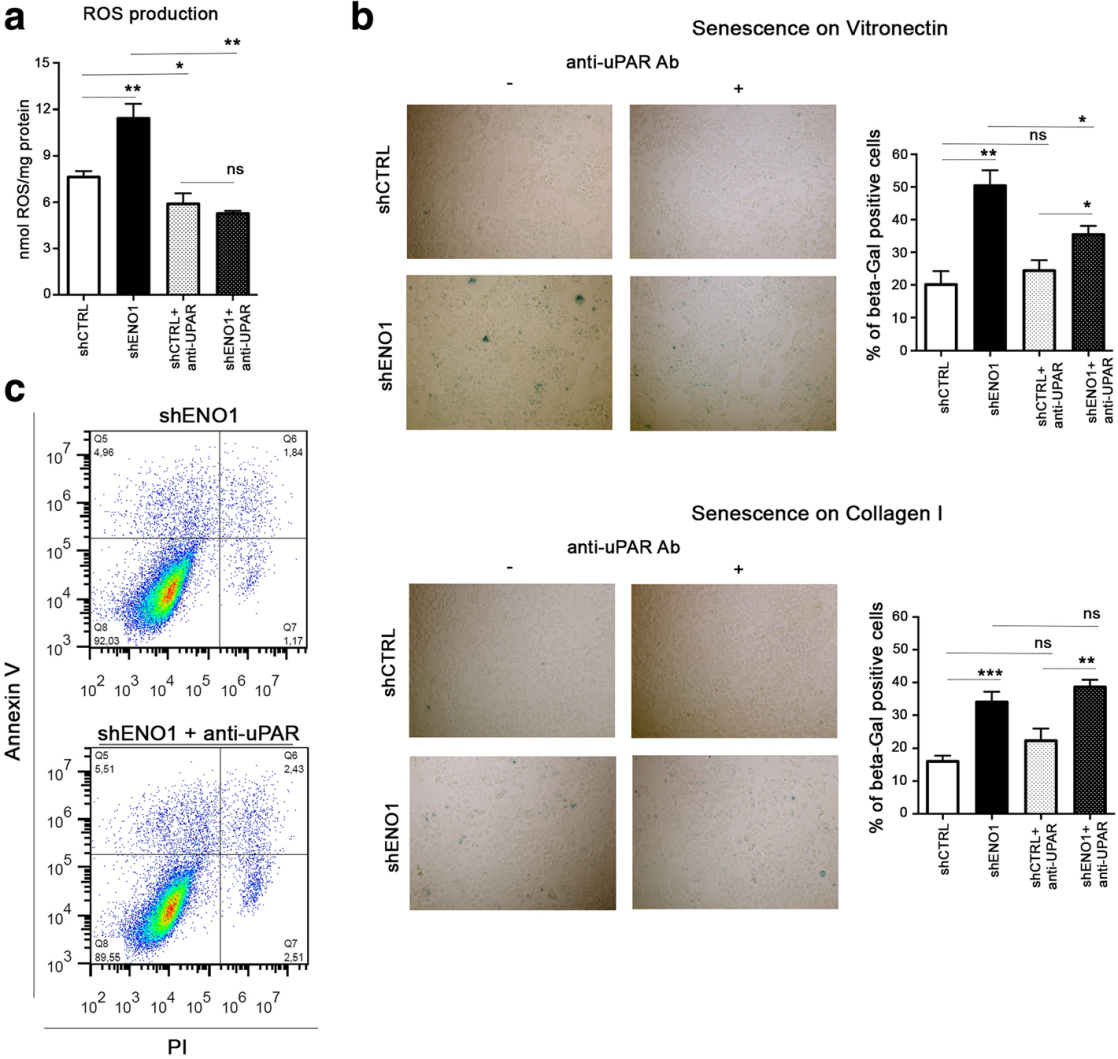
Analysis of reactive oxygen species (ROS) production, senescence, and apoptosis in shENO1 cells lines after anti-uPAR Ab treatment. **a** Analysis of ROS concentration measured by the DCFDA-AM assay was evaluated in shENO cells and control cells in the presence or absence of anti-uPAR antibody. **b** Senescence-associated β-galactosidase staining. Senescent shENO and shCTRL cells were colored blue upon X-gal staining at pH 6, with or without treatment with anti-uPAR antibody. One representative out of three independent experiments is shown. The graph represents the percentage of X-Gal positive cells with respect to the total number of cells. **c** Dot plot of shENO1 cells apoptotic cells in the presence or absence of anti-uPAR antibody. Apoptotic cells were evaluated as early apoptotic (AnxV pos/ PI negative) + late apoptotic cells (AnxV pos/ PI positive). Data are mean ± SEM. *p < 0.05, **p < 0.01, ***p < 0.001 relative to control cells

Our previous work demonstrated that ENO1 silencing increased reactive oxygen species (ROS) mainly generated through the sorbitol and NADPH oxidase pathways, which affect cancer cell growth and induce senescence [19]. As ERK1-2/RAC activation leads to an increase of ROS and senescence [37], we investigated if uPAR is also involved in these phenomena. ROS were analyzed by measuring intracellular 5-(and-6)-chloromethyl- 2′,7′-dichorodihydro-fluorescein diacetate (DCFDA) fluorescence. Treatment with the anti-uPAR antibody blocked the binding of uPAR to beta 1 integrin and specifically reduced the production of ROS in shENO1 cells, but not in shCTRL control cells (Fig. 6a).

As uPAR directly binds vitronectin, we observed that the anti-uPAR blocking antibody inhibited senescence of shENO1 cells plated on vitronectin (Fig. 6b upper panel), but not that of shENO1 cells plated on collagen I (Fig. 6b lower panel) suggesting that the senescence can be induced by VN/uPAR downstream effects. The anti-uPAR blocking antibody slightly increased the apoptosis of shENO1 PDA cells (Annexin V positive cells Fig. 6c).

### ENO1 silencing impairs in vitro and in vivo PDA cell migration and invasion

**Fig. 7:**
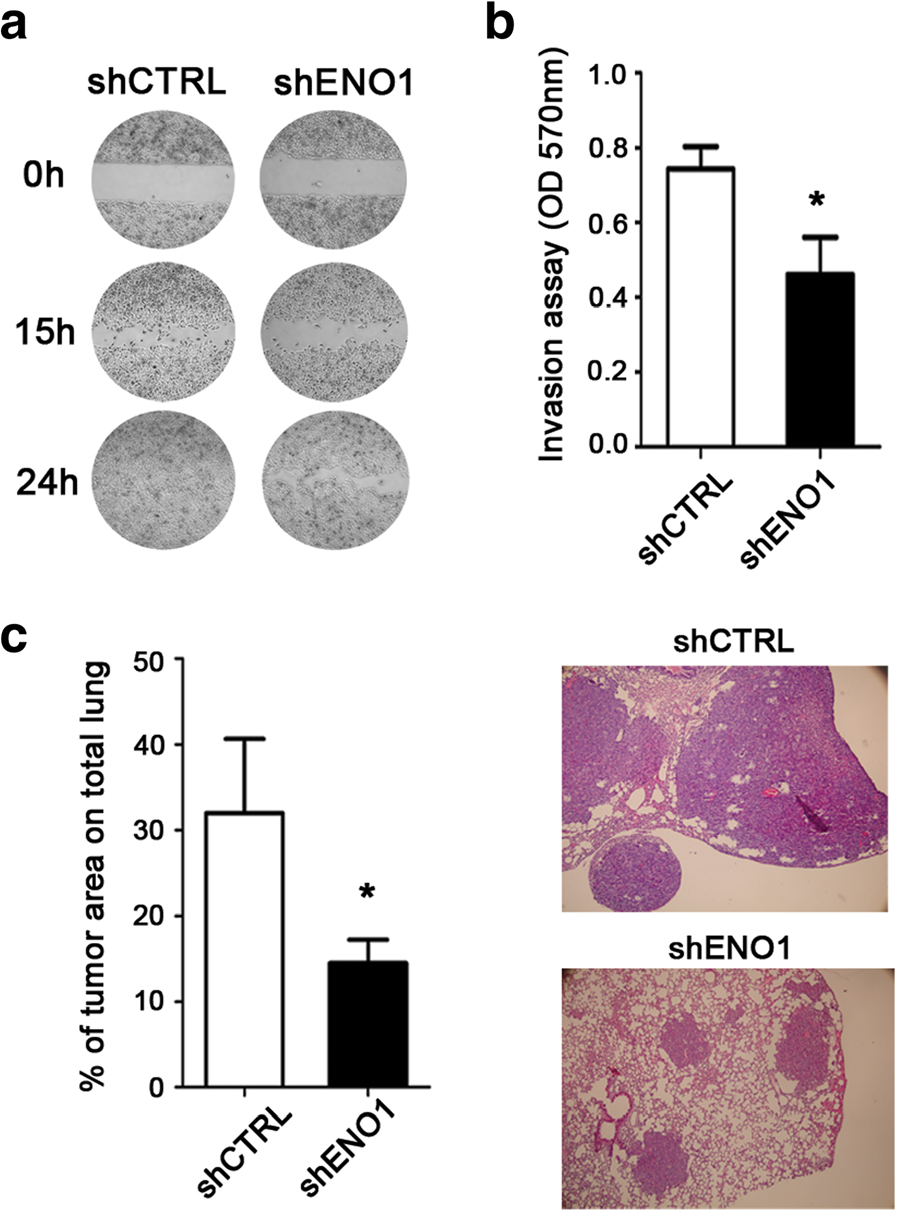
ENO1 silencing affects in vitro and in vivo cell migration and invasion. **a** Migration ability was evaluated in terms of capacity to close the wound of shENO1 CFPAC-1 cells compared to shCTRL control cells. Representative images are shown for each condition. **b** Invasive potential of shENO1 and shCTRL cells were tested through a Matrigel invasion assay, after 48 h of culture. For quantitative analysis, invasive cells were fixed, stained with crystal violet, treated with acetic acid and elutes were read at a wavelength of 570 nm. Results are expressed as OD. **c** Lung metastatic area was evaluated after 28 days from i.v. injection of shCTRL or shENO1 CFPAC-1 cells in the tail vein of NSG mice. The histogram represents the percentage of metastatic area compared to the total lung (*left panel*). Representative images of the lung are shown for each group of mice (*right panels*). For the in vivo experiments, five mice per group were used. A representative of three independent experiments is shown. Data are mean ± SEM. *p < 0.05 relative to control cells

Considering the cytoskeleton and integrin pathway alterations in shENO1 cells, we investigated their migration and invasiveness by wound-healing scratch and Matrigel cell invasion assays, respectively. While control cells resulted in more than 60–80% of the wound being closed at 15 h, and 100% after 24 h, there was no wound-healing evident on the shENO1 cell lines (CFPAC-1, PT45, and T3M4), even after 24 h (Fig. 7a and Additional file 1: Figure S1c). Cell invasion through Matrigel also demonstrated a significantly lower ability of shENO1 cell lines to invade compared to control cells (Fig. 7b and Additional file 1: Figure S1d).

To assess the effect of ENO1 silencing in metastasis formation in vivo, shENO1 or shCTRL CFPAC-1 cells were injected intravenously into NSG mice. After 28 days following injection, mice were sacrificed and lungs were excised and checked for the presence of metastasis. Post-mortem observations confirmed that injection with shENO1 cells resulted in a significant reduction of the tumor area in the lungs compared to mice injected with control cells (Fig. 7c). These data confirmed that changes in the adhesion, migration, and invasion ability observed in vitro in shENO1 cells markedly compromised their ability to spread and form metastasis in vivo, demonstrating that ENO1 may exert a crucial role in invasion and metastasis.

## Discussion

In our previous report, we demonstrated that ENO1 cell surface expression is important for plasminogen-dependent invasion, and that targeting of ENO1 with a monoclonal antibody inhibits the invasiveness of pancreatic cancer cells [12]. These data correlate well with previous observations in follicular thyroid carcinoma [38], glioma [13], non-small cell lung cancer [14], and endometrial cancer [15], again supporting the emerging role of ENO1 as a promising target for cancer treatment. However, although blocking surface ENO1 impaired the ability of PDA cells to migrate in vitro and form more metastasis in vivo [12], little is known about the role of ENO1 in regulating cell-cell and cell-matrix contacts. Proteomic analysis of silenced ENO1 in PDA cells revealed a profound modification in their metabolism, which was associated with an increase of oxidative stress and senescence [19]. In addition, although silenced PDA cells also displayed alterations in many molecules involved in adhesion, as well as cytoskeletal proteins, this study aimed to investigate more thoroughly the role of ENO1—a multifunctional protein involved in cell-matrix adhesion, motility and migration, invasion and metastasis in vivo, as well as in survival and senescence.

By employing a combination of confocal and AFM-assisted nanostructural approaches, we investigated the phenotype and morphology of the PDA cells in the presence and absence of ENO1. Profound morphological changes in shENO1 PDA cells were observed, due to the modification of the cytoskeleton organization. Control cells exhibited smooth topography, while the shENO1 cells displayed a rough surface. The increase in surface roughness was consistent with the topographical changes observed with the AFM images. Cell nanostructural parameters, such as elasticity or roughness, may reflect a reorganization of the actin cytoskeleton, which in turn affects cell growth, morphology, cell-cell interactions, cytoskeleton organization, and interactions with the ECM [39]. It is known that ENO1 knockdown induces a dramatic increase of the sensitivity to microtubule-targeted drugs (e.g., taxanes and vincristine) in different tumor cell lines, due to ENO1-tubulin interactions [40], suggesting a role for ENO1 in modulating the cytoskeletal network. Consistently, we demonstrated that ENO1 critically contributed to the organization of the actin cytoskeleton. Indeed, in control cells, actin was organized into filaments that were mostly distributed close to the cell surface, while in shENO1 cells, the organization of the actin filaments was lacking, and actin prevalently relocalized close to the nuclei. This actin modification suggested a profound reorganization of cytoskeleton regulatory proteins, suggesting that ENO1 silencing reduced the ability of PDA cells to adhere to ECM and migrate. This is in agreement with our previous proteomic analysis, which showed that ENO1 silencing downregulated many proteins involved in motility pathways [19].

Consistent with the proteomic data, shENO1 PDA cells showed a decreased adhesion to FN and Cols, and a reduction in migration and invasion. Of the proteins downregulated after ENO1-silencing, we highlight alpha v/beta 3 integrin, a crucial protein involved in spreading and metastasis, the increased expression of which correlates with a poor clinical outcome in PDA [21–23]. FN is recognized by either alpha 5/beta 1 or alpha v/beta 3 complexes, which cooperate in promoting cellular attachment and spreading [41]. Our data show that ENO silencing downregulates alpha v/beta 3 but increases alpha 5/beta 1 expression. Considering that alpha 5/beta 1 determines adhesion strength through its binding to FN, while alpha v/beta 3 mediates reinforcement of the adhesion through its connection with the actin cytoskeleton [42, 43], we speculate that in shENO1 cells, while the alpha 5/beta 1 complex begins the adhesion process, there is a reduced adherence to FN due to the lack of reinforcement signals controlled by the alpha v/beta 3 complex. Surprisingly, ENO1 silencing is also associated with an increase of adhesion to VN. In cancer, VN interacts with different members of the integrin family (alpha v/beta 1, alpha v/beta 3, alpha v/beta 5, and alpha IIb/beta 3) through the RGD motif [44, 45]. As alpha v and beta 3 subunits were shown to be downregulated in shENO1 cells, none of the abovementioned complexes can be considered to be responsible for the increased adhesion of shENO1 cells to VN. This suggests that a non-integrin receptor is involved in the binding of VN. As uPAR binds VN through a different binding site from that of integrins [34, 46, 47], we hypothesized that uPAR plays a major role in VN binding in shENO1 cells.

uPAR expression is elevated in many human cancers, in which it frequently indicates poor prognosis [48]. uPAR regulates proteolysis by binding to the extracellular protease urokinase-type plasminogen activator (uPA) [49] and also activates many intracellular signaling pathways [50]. Exploiting the above functions, uPAR regulates important functions such as cell migration, proliferation, and survival, and thus makes it an attractive therapeutic target in various different types of cancer [36]. Here, we observed the upregulation of uPAR in shENO1 cells, which have a reduced ability to invade and form metastases, and an increased senescence, suggesting that uPAR may contribute in a different way in this setting. uPAR, lacks a cytosolic domain, and thus signals through its association with integrins, which can also be independent of direct integrin/matrix interactions, in a ligand-independent manner, to promote migration [34]. The major downstream uPAR/integrin signaling (especially beta 1 and beta 3) involves the activation of Src, PI3K/AKT, and MEK/ERK1-2 pathways [50]. In shENO1 PDA cells we observed the activation of Src. The effect of increased Src activity in cells is pleiotropic [51]. In particular, cells with activated Src are characterized by a loss of actin reorganization and reduced cell-ECM adhesion [51], phenomena that perfectly match our observations.

In NSCLC, the downregulation of ENO1 decreased proliferation, migration, and invasion through a FAK-mediated PI3K/AKT pathway [14]. Clustering of integrins activates FAK results in the formation of a complex with Src, and increased phosphorylation of the targets of the FAK-Src complex, such as paxillin [52]. In pancreatic cancer we did not observe an altered phosphorylation of FAK and Paxillin as well as AKT. Instead, in our study, we observed that ENO1 silencing leads to uPAR overexpression that, in turn, triggers Src and ERK1-2 activation, concomitantly with an inactivation of p38MAPK. The activation of ERK1-2 can promote senescence, in accordance with Cagnol et al. [53]. Conversely, activation of p38MAPK can promote apoptosis in cancer cells, including pancreatic cancer [54]*.* We observed that shENO1 cells, due to the decrease in phosphorylation of p38MAPK concurrently with ERK1-2 activation, slightly inhibit PDA cell apoptosis and favor senescence, in line with our previously reported results [19].

ERK1-2 activation, due to the uPAR-beta 1 integrin interaction is required for RAC activation [36, 55–59]. RAC is a downstream signaling molecule of beta 1 and contributes to the regulation of actin cytoskeleton dynamics, adhesion, and migration and induces cellular reactive oxygen species (ROS) through NAPDH oxidase activation [37]. Expression of the constitutively active RAC1 mutant induces cell cycle arrest, apoptosis, and senescence [37]. We have previously demonstrated that ENO1 silencing induces ROS, mainly through the sorbitol and NADPH oxidase pathway and senescence [19]. Here we demonstrate that, in the absence of ENO1, the upregulation of uPAR leads to an increased activation of the ERK1-2/RAC pathway, which contributes to ROS generation and induces PDA cell senescence, rather than an invasive phenotype. Moreover, the anti-uPAR antibody prevented ROS production and senescence although PDA cell apoptosis was only slightly promoted. These results suggest that a combinatory strategy to simultaneously target ENO1 and uPAR could be effective to inhibit PDA tumor progression and invasion.

## Conclusions

Our study has shown, by in vitro and in vivo experiments, that ENO1 silencing can inhibit adhesion, invasion, and metastasis in PDA cells, due to changes in actin cytoskeleton organization, adhesion proteins, and integrin profile expression. ENO1 silencing had a major impact on the alpha v/beta 3 integrin, which accounts for the inability of ENO1-silenced cells to adhere to the ECM matrix and promote PDA invasion. We have reported that, in the absence of ENO1, the upregulation of uPAR does not promote an increase of migration or invasion. These data show that there is an interplay of ENO1 with integrins and uPAR, which critically controls PDA progression.

## Additional file

Additional file 1: Table S1. Oligonucleotide primer sequences for SybrGreen qRT-PCR. **Figure S1a–d** Adhesion, migration and invasion ability of PT45 and T3M4 shENO1 PDA cells. Adhesive potential of shENO1 PT45 (a) and shENO1 T3M4 (b) compared to relative shCTRL control cells was evaluated by culturing cells for 1 h on FN, Col-I, Col-IV and VN. Adherent cells were fixed with 2% glutaraldehyde in PBS and visualized by staining with crystal violet. For quantitative analysis, cells were treated with 10% acetic acid and elutes were read with a microplate reader at a wavelength of 570 nm. Results are expressed as Δ ^⋿^ OD (Optical Density) units = (OD substrate adherent cells) – (OD plastic adherent cells). (c) Migration ability was evaluated in terms of ability to close the wound with shENO1 PT45 and shENO1 T3M4 cells compared to shCTRL control cells. Representative images are shown for each condition. (d) Invasive potential of shENO1 PT45 and shENO1 T3M4 were tested by a Matrigel invasion assay, after 48 h of culture. For quantitative analysis, invasive cells were fixed, stained with crystal violet, treated with acetic acid and elutes were read at a wavelength of 570 nm. Data are mean ± SEM of at least three independent experiments. *p < 0.05, **p < 0.01, ***p < 0.001 (DOCX 326 kb)

## Abbreviations

Ab: Antibody
AFM: Atomic force microscopy
AGR2: Anterior gradient protein 2
AHNAK: AHNAK nucleoprotein isoform 1
AnxV: Annexin V
APC: Allophycocyanin
ARPC4: Actin related protein 2/3 complex subunit 4 isoform a
ATP: Adenosine triphosphate
BCAR1: Breast cancer anti-estrogen resistance 1
CAPZA2: Capping protein actin filament muscle Z-line alpha 2
CAT: Confocal–atomic force microscopy–total internal reflection fluorescence
Col: Collagen
CRIP1: Cysteine-rich protein 1 intestinal
CTNNA1: Catenin alpha 1
CTNND1: Catenin, delta 1 isoform 1ABC
ECM: Extracellular matrix
ENO1: Alpha-enolase
ERK1-2: Extracellular signal–regulated kinases 1–2
FAK: Focal adhesion kinases
FN: Fibronectin
GLG1: Golgi apparatus protein 1 isoform 1
GST: Glutathione S-transferase
HRP: Horseradish peroxidase
IgG: Immunoglobulin G
ITGAV: Integrin alpha v isoform 1 precursor
LC-MS/MS: Liquid chromatography-mass/mass spectrometry
LGALS3: Galectin 3
LGALS4: Galectin 4
MAPK: Mitogen-activated protein kinase
MINERVA: Hypothetical protein LOC64855 isoform 2
MUC5AC: Mucin 5AC
NSG: NOD/SCID gamma
Pax: Paxillin
PBS: Phosphate-buffered saline
PCR: Polymerase chain reaction
PDA: Pancreatic ductal adenocarcinoma
PDLIM1: PDZ and LIM domain 1
PI: Propidium iodide
RGD: Arg-Gly-Asp
ROS: Reactive oxygen species
SA-beta-Gal: Senescence associated beta-Galactosidase
SEM: Standard error of the mean
SERPINb5: Serine or cysteine proteinase inhibitor clade B ovalbumin member 5
shCTRL: Control cell line
shENO1: ENO1-silenced cell line
shRNA: Short hairpin RNA
SMB: Somatomedin B
SPP1: Secreted phosphoprotein 1 isoform a
TRITC: Tetramethylrhodamine B isothiocyanate
uPAR: Urokinase plasminogen activator receptor
VN: Vitronectin
