# Supplementary material for: Alpha-enolase (ENO1) controls alpha v/beta 3 integrin expression and regulates pancreatic cancer adhesion, invasion, and metastasis
Source: J Hematol Oncol. 2017 Jan 13;10:16. doi: 10.1186/s13045-016-0385-8 (PMC5237223; doi:10.1186/s13045-016-0385-8)
Supplement: Additional file 1: Table S1. — Oligonucleotide primer sequences for SybrGreen qRT-PCR. Figure S1a–d Adhesion, migration and invasion ability of PT45 and T3M4 shENO1 PDA cells. Adhesive potential of shENO1 PT45 (a) and shENO1 T3M4 (b) compared to relative shCTRL control cells was evaluated by culturing cells for 1 h on FN, Col-I, Col-IV and VN. Adherent cells were fixed with 2% glutaraldehyde in PBS and visualized by staining with crystal violet. For quantitative analysis, cells were treated with 10% acetic acid and elutes were read with a microplate reader at a wavelength of 570 nm. Results are expressed as Δ ⋿ OD (Optical Density) units = (OD substrate adherent cells) – (OD plastic adherent cells). (c) Migration ability was evaluated in terms of ability to close the wound with shENO1 PT45 and shENO1 T3M4 cells compared to shCTRL control cells. Representative images are shown for each condition. (d) Invasive potential of shENO1 PT45 and shENO1 T3M4 were tested by a Matrigel invasion assay, after 48 h of culture. For quantitative analysis, invasive cells were fixed, stained with crystal violet, treated with acetic acid and elutes were read at a wavelength of 570 nm. Data are mean ± SEM of at least three independent experiments. *p < 0.05, **p < 0.01, ***p < 0.001 (DOCX 326 kb) [file 13045_2016_385_MOESM1_ESM.docx]

**SUPPL. Table 1.** Oligonucleotide primer sequences for SybrGreen qRT-PCR

| ENO f | GCCTCCTGCTCAAAGTCAAC |
| --- | --- |
| ENO r | AACGATGAGACACCATGACG |
| ARPC4 f | ACCTGTGACCATCAGCAGGAATGA |
| ARPC4 r | TCATGAAGCGCATGAACTTGTGGC |
| CAPZA2 f | GTGTGCGGTTACAGTCTAAAT |
| CAPZA2 r | ACTCCACTTCACTCCCTATG |
| SPP1 f | CCACAAGCAGTCCAGATTAT |
| SPP1 r | GGGTCTACAACCAGCATATC |
| BCAR1 f | AGGAGGAGTTTGAGAAGACCCAGA |
| BCAR1 r | ACACCTCCTGTTCCAGTCGTTCAA |
| AHNAK f | TCCAGGCACCTGATCTGGAACTTT |
| AHNAK r | ACATCTGGCATCTTGACCTTGGGA |
| AGR2 f | TAAATGCAGCACTAGTGGGTGGGA |
| AGR2 r | AGGAGCAAGAATGCTGACACTGGA |
| CTNND1 f | TACCAAGAGGCAGCTCCCAATGTT |
| CTNND1 r | TGGAGAACCACTCATCTTTGCCCT |
| MINERVA f | TGCGCGTCACTACTACTTCTGCAT |
| MINERVA r | ATTCCATTGTTGCAGTGCCGGATG ATTCCATTGTTGCAGTGCCGGATG |
| LGALS3 f | GTGCCTTATAACCTGCCTTTGCCT |
| LGALS3 r | TTCTGTTTGCATTGGGCTTCACCG |
| CTNNA1 f | AGCTTGTTCGAATGTCTGCAAGCC |
| CTNNA1 r | ACAGCATCTGTGAGAACACGGACT |
| ITGAV f | TAGCAACTCGGACTGCACAAGCTA |
| ITGAV r | AACCATTCCCAAAGTCCTTGCTGC |
| LGALS4 f | CTTCGTGAACTTTGTGGTTGGGCA |
| LGALS4 r | TTTGAAGGGCATGCTCCTCTTCCT |
| GLG1 f | AGGTGAGCTGTCTGGGCAAACTAT |
| GLG1 r | AGCAATGGAAGTCCAACATGCACC |
| MUC5AC f | CTCCTACAACGAGCACAAC |
| MUC5AC r | ACAGGGCTGGGAGTATTT |
| SERPINb5 f | CTCACGTTACCTTGACACATAG |
| SERPINb5 r | GAACCTGCAACACGGATAG |
| PDLIM1 f | AAGTGGCTGCGTCGATTGGAAATG |
| PDLIM1 r | CGCAGCTTCACAAACACACCAACA |
| CRIP1 f | AGAAATGTGGGAAGACGCTGACCT |
| CRIP1 r | TACTTGAAAGTGTGGCTCTCGGCT |
| AlphaIIb f | CACTGAATCCTGCTGTGAAGA |
| AlphaIIb r | CAGCTCGGCATTTAGGGATAG |
| Beta3 f | CATCCATAGCACCTCCACATAC |
| Beta3 r | CCAGCCAACTCATGGGAATAA |
| Alpha 5 f | GGACTGTGGAGAAGACAACATC |
| Alpha 5 r | GTGAGGTTCAGGGCATTCTT |
| Beta1 f | ATCCCATTGACCTCTACTACCT |
| Beta1 r | GTCCGAAGTAATCCTCCTCATTT |
| uPAR f | AGCTGTACCCACTCAGAGAA |
| uPAR r | CCCTGGTTGCACAAGTCTAA |
| b-ACT f | CGCCGCCAGCTCACCATG |
| b-ACT r | CACGATGGAGGGGAAGACGG |

“f”: forward; “r”: reverse


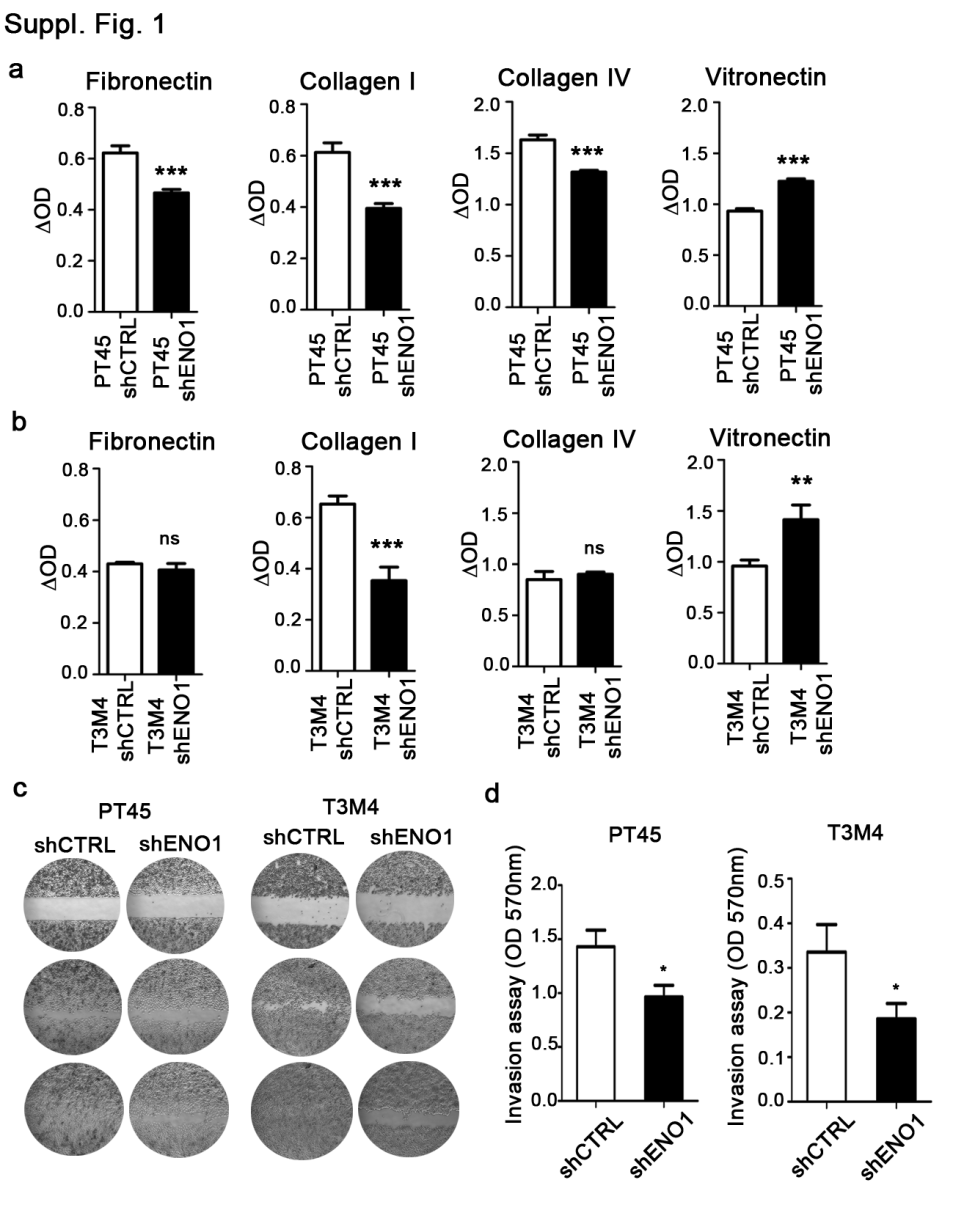


**Supplementary Figure 1. Adhesion, migration and invasion ability of PT45 and T3M4 shENO1 PDA cells.** Adhesive potential of shENO1 PT45 **(a)** and shENO1 T3M4 **(b)** compared to relative shCTRL control cells was evaluated by culturing cells for 1 h on FN, Col-I, Col-IV and VN. Adherent cells were fixed with 2% glutaraldehyde in PBS and visualized by staining with crystal violet. For quantitative analysis, cells were treated with 10% acetic acid and elutes were read with a microplate reader at a wavelength of 570 nm. Results are expressed as ΔOD (Optical Density) units = (OD substrate adherent cells) – (OD plastic adherent cells). **(c)** Migration ability was evaluated in terms of ability to close the wound with shENO1 PT45 and shENO1 T3M4 cells compared to shCTRL control cells. Representative images are shown for each condition. **(d)** Invasive potential of shENO1 PT45 and shENO1 T3M4 were tested by a Matrigel invasion assay, after 48 h of culture. For quantitative analysis, invasive cells were fixed, stained with crystal violet, treated with acetic acid and elutes were read at a wavelength of 570 nm. Data are mean ± SEM of at least three independent experiments. *p < 0.05, **p < 0.01, ***p < 0.001
